# Seasonal changes in invertebrate diet of breeding black‐necked cranes (*Grus nigricollis*)

**DOI:** 10.1002/ece3.70234

**Published:** 2024-08-29

**Authors:** Ruifeng Ma, Shujuan Ma, Hongyi Liu, Lei Hu, Yudong Li, Ke He, Ying Zhu

**Affiliations:** ^1^ College of Grassland Resources, Institute of Qinghai‐Tibetan Plateau, Sichuan Provincial Forest and Grassland Key Laboratory of Alpine Grassland Conservation and Utilization of Qinghai‐Tibetan Plateau Southwest Minzu University Chengdu Sichuan China; ^2^ The Co‐Innovation Center for Sustainable Forestry in Southern China, College of Life Sciences Nanjing Forestry University Nanjing China; ^3^ Sichuan Province Laboratory for Natural Resources Protection and Sustainable Utilization Sichuan Provincial Academy of Natural Resource Sciences Chengdu China; ^4^ College of Animal Science and Technology, College of Veterinary Medicine Zhejiang A&F University Hangzhou China

**Keywords:** breeding black‐necked crane, DNA metabarcoding, invertebrate, marsh bird, seasonal dietary variation

## Abstract

Invertebrates greatly support the growth, development, and reproduction of insectivorous birds. However, the influence of human activity (e.g., pesticide use, deforestation, and urbanization) inevitably leads to a decrease in global arthropods. The diversity and variation in invertebrate diet influence the food composition of birds, especially species living in rapidly changing environments, such as the Tibetan Plateau. However, little is known of the seasonal variation in invertebrate diet in response to environmental changes. Here, we characterized the invertebrate diet composition in pre‐ and post‐breeding black‐necked crane (*Grus nigricollis*) using fecal metabarcoding. We identified 38 invertebrate genera; the top three were *Tipula* (82.1% of relative abundance), *Ceramica* (3.0%), and *unclassified_Hymenoptera* (*2.5%*), with *Tipula* predominated the diet in both seasons. We also observed 20 and 16 unique genera in the pre‐ and post‐breeding periods, and the genera composition was distinct between seasons (*R* = .036, *p* = .024). In pre‐breeding, black‐necked cranes tended to consume more diverse foods, and individual cranes exhibited greater heterogeneity at the genus level. At the genera and species level, pre‐breeding black‐necked cranes showed a wider dietary niche than post‐breeding cranes. We observed season‐specific features, with *Tipula* (common crane fly) and *Stethophyma* (grasshoppers) being enriched in the post‐breeding period and *Ceramica* (moth) being more abundant in the pre‐breeding period. Three *Tipula* species had the greatest importance in discriminating between seasonal diets. This study demonstrated a seasonal pattern of invertebrate diet in the black‐necked crane, suggesting diet composition in response to resource and species availability. These results elaborate on the foraging ecology of highland birds and can inform the management of black‐necked crane conservation.

## Graphical abstract

The growth, development, and reproduction of birds are influenced by the invertebrate diet. We studied the feeding composition of invertebrates in pre‐ and post‐breeding of black‐necked cranes (*Grus nigricollis*) by using fecal metabarcoding. We found that black‐necked cranes had a wider dietary niche at the species level in pre‐breeding, and we observed season‐specific features. This study demonstrated a seasonal pattern of invertebrate diet in the black‐necked crane, suggesting diet composition in response to resource and species availability. These results elaborate on the foraging ecology of highland birds and can inform the management of black‐necked crane conservation.

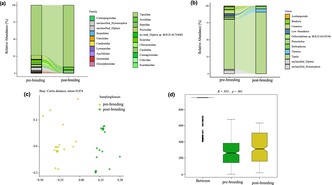

## 1 INTRODUCTION

Invertebrates, the most diverse animal group, are a critical resource in the growth, development, and reproduction of insectivorous birds (Józefiak et al., 2016; Reeves et al., 2021, 2023), and constitute a substantial proportion of their dietary intake (McClenaghan et al., 2019; Nell et al., 2023; Stillman et al., 2022), mainly owing to their high nutritional value (e.g., high protein and fat/energy contents) compared to plant‐based resources (Day et al., 2022; Ewy et al., 2022; Gazzani et al., 2019).

It has been reported that some human activities (e.g., pesticide use, deforestation, and urbanization) lead to a decrease in global arthropods (Kwon et al., 2013; Raupp et al., 2010). The decrease in arthropods might reduce nutrition intake, possibly affecting the number of insect‐eating birds (Otieno & Mukasi, 2023; Schmidt et al., 2005). Studies have observed that the decline in bird population might be related to arthropods decline, highlighting the importance of arthropods in birds' diet (Nell et al., 2023; Sotherton & Self, 2000). Consequently, describing the diversity and variation in the invertebrate diet of birds could help identify their adaptations to surviving environmental changes and inform the conservation of threatened bird species.

Insectivorous birds exhibit interspecific, temporal, and spatial variations in invertebrate diet composition (Murphy et al., 2023; Vonshak et al., 2009). Seasonal changes affect dietary variation in many species, such as Sichuan partridges (*Arborophila rufipectus*) (Tang et al., 2023), wintering ducks (such as *Anas acuta*, *Spatula clypeata*, and *Mareca penelope*) (Ando et al., 2023), and Eurasian otter (*Lutra lutra*) (Martínez Abraín et al., 2020). These seasonal effects are closely related to changes in resource availability (Hou et al., 2021; Spitzer et al., 2020). The Tibetan Plateau, known as the “Third Pole,” is the largest, highest plateau on earth, and its harsh climatic conditions have led to a relatively fragile ecological environment, where vegetation is extremely vulnerable to the effects of environmental climate change (Che et al., 2014). Moreover, the Tibetan Plateau has substantial temperature and precipitation differences between the non‐growing and growing seasons (Du et al., 2016; Shen et al., 2022). Therefore, birds of the Tibetan Plateau are prone to experiencing extreme shifts in resource availability, resulting in seasonal dietary variation. However, studies on seasonal diets are limited to a few species, such as the Eurasian tree sparrow (*Passer montanus*) (Sun et al., 2023) and some raptor species (e.g., Eurasian eagle owl [*Bubo bubo*], Saker falcon [*Falco cherrug*]) (Hacker et al., 2021) in high altitudes.

The black‐necked crane (*Grus nigricollis*) is a near‐threatened insectivorous species breeding and overwintering on plateaus (2500–5000 m above sea level) (Song et al., 2014; Zhao et al., 2021). They inhabit Qinghai‐Tibet Plateau wetlands during the breeding season (May–September) and migrate to the Yunnan‐Guizhou Plateau for winter (Yang et al., 2007). The black‐necked crane population was drastically reduced due to habitat loss before 2012 (Fang et al., 2020) but has been gradually increasing owing to habitat protection projects2024 (Wu et al., 2024). Typically, black‐necked cranes nest in approximately May and use a two‐parent rotation to incubate their eggs (Yuan et al., 2023). Their breeding areas are relatively scattered, they typically prefer swamps and nest islands in lakes and other freshwater bodies to protect their eggs from predators, and they forage in shallow water (Song et al., 2014), just like other cranes, such as sandhill crane (*Grus canadensis*) (Krapu et al., 2019) and siberian crane (*Grus leucogeranus*) (Harris & Mirande, 2013). Different from breeding cranes, farmland is the major foraging ground other than wetlands during winter (Yuan et al., 2023).

Previous studies on fecal micromorphology, direct observations, and video analysis revealed that the black‐necked crane feeds on a limited variety of arthropods during the overwintering period (Dong et al., 2016), while it mainly feeds on Coleoptera, Hymenoptera, and Diptera during the breeding period (Liu et al., 2019). Because animal food is easily digestible (Sá et al., 2020), little is known about the dietary composition of invertebrates in black‐necked cranes (mainly at the family and genus levels). During the breeding period, migratory birds are faced with variable energy demands; changes in feeding strategies can reflect adaptations to energy demands (Dunn et al., 2020; Marn et al., 2022). However, few studies have investigated diet changes in black‐necked cranes before and after breeding.

DNA metabarcoding (based on high‐throughput sequencing) provides a non‐invasive and high‐resolution technique (e.g., more food items detected and more accurate taxonomy assignment) for determining the complex dietary composition in herbivorous, carnivorous, and omnivorous species compared to direct observation, gastrointestinal dissection, or fecal micromorphology (De Barba et al., 2014; de Sousa et al., 2019; Sow et al., 2020; Thuo et al., 2019). Traditional methods, such as microscopic examination, are easily affected by food digestion, resulting in some food being difficult to identify. For example, major diets were four times more likely to be detected by DNA than by microscopic examination in wild boar (*Sus scrofa*) (Monterroso et al., 2019). In hoofed animals, an average of 90% of DNA sequences were identified to the genus or species level, compared with the 75% of plant fragments detected using microscopy. Based on the aforementioned advantages, the DNA metabarcoding technique is now widely used for food identification in insectivorous birds such as common cranes (*Grus grus*) (Zhao et al., 2023), Red‐crowned Cranes (*Grus japonensis*) (Liu, Xu, et al., 2023), and demoiselle cranes (*Grus virgo*) (Li et al., 2024).

In this study, we investigated the invertebrate diet composition of breeding black‐necked cranes based on DNA metabarcoding. We aimed to determine the (1) diet composition of fine‐scale invertebrates during the breeding period in highland alpine wetlands and (2) seasonal dietary variation between spring (pre‐breeding) and autumn (post‐breeding). This comprehensive and seasonal analysis elaborates on the role of invertebrates in bird diet, the adaptations of birds to high‐altitude environments, and could inform the conservation of threatened insectivorous highland birds.

## 2 MATERIALS AND METHODS

### 2.1 Study area and fecal sample collection

The study area was located in the Zoige National Reserve, in the eastern part of the Tibetan Plateau. This is the largest highland peatland globally and is characterized as a typical highland alpine wetland ecosystem (Bai et al., 2022). We researched the specific foraging area of black‐necked cranes during the breeding season by conducting a comprehensive literature review (Dou et al., 2013; Fang et al., 2020; Jiang et al., 2017), interviews, and surveys conducted with local herders, and consultation with the wetland reserve staff.

**FIGURE 1:**
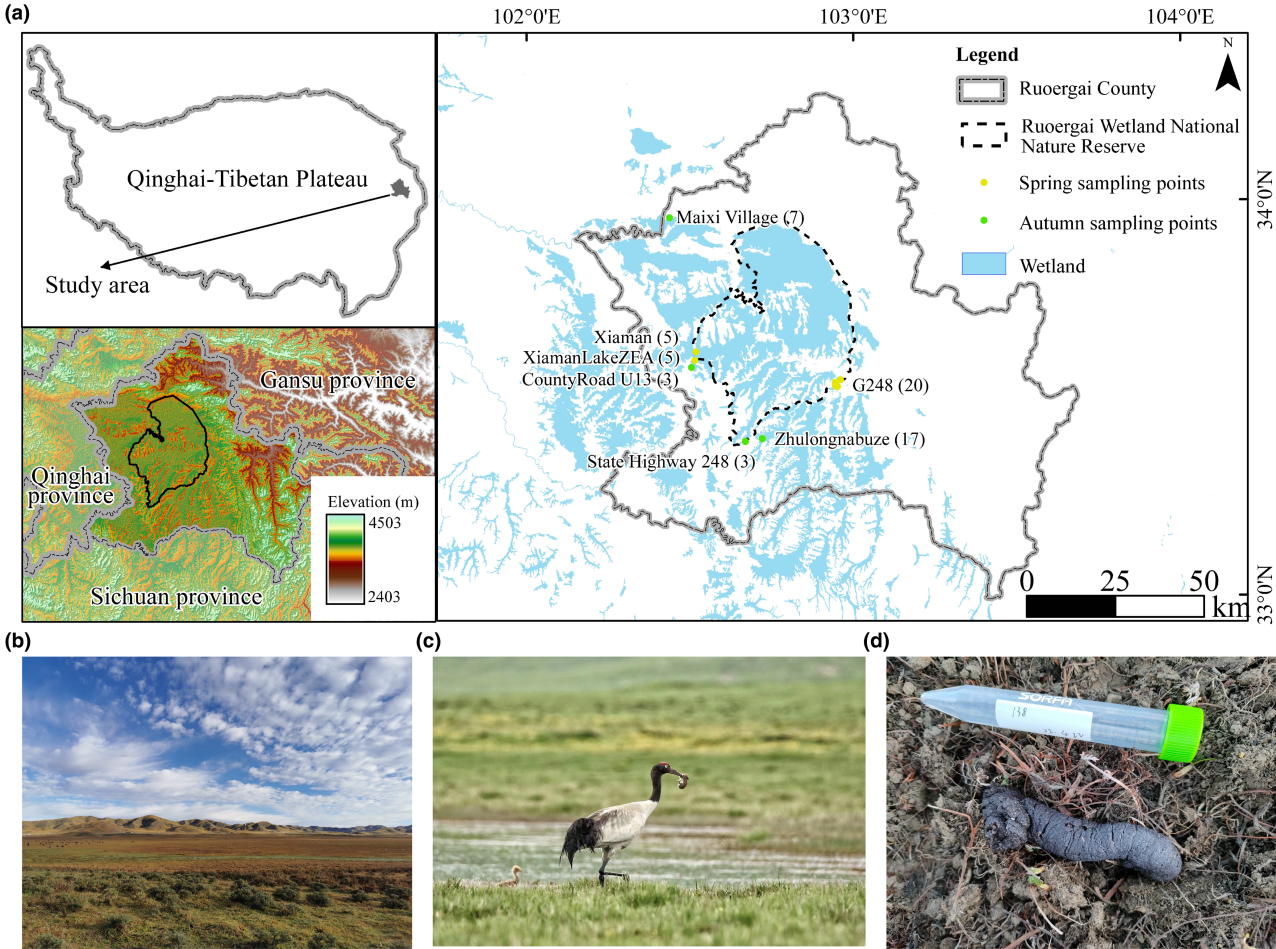
Study site and sampling locations. (a) Sampling sites in Zoige Wetland National Nature Reserve. The numbers in parentheses indicate the corresponding sample sizes. (b) Foraging landscape. (c) Black‐necked crane in breeding area. (d) Black‐necked crane droppings.

We searched for and observed the foraging behavior of any located flocks. Once the cranes departed (after ~2 h), we visited the foraging area to search for fresh fecal samples. To prevent collecting multiple samples from the same crane, we collected feces at >5 m intervals. After removing the outer layer of fecal samples using a sterilized toothpick and disposable PE gloves, the fecal matter was transferred into 15 mL sterile centrifuge tubes, stored temporarily in an icebox, and transported to the research facility. The fecal samples were then preserved in liquid nitrogen and stored at a −80°C. The pre‐ and post‐breeding periods were in April and September 2022, respectively. Overall, we collected 60 fecal samples from 11 sites, 30 samples for each time point, during the pre‐ and post‐breeding periods (Figure 1, Table S1, Figure S1). We extracted DNA from the internal part of the fecal sample to avoid contamination from the environment.

### 2.2 Species identification in fecal samples

Feces were grinded in a sterilized mortar with liquid nitrogen prior to DNA extraction using a Tiangen Magnetic Soil and Stool DNA Kit (Beijing, China), following the manufacturer's instructions. We designed two nested pairs of cytB primers for species identification based on the mitochondrial genome sequences of 15 crane species. The first round of PCR primers included CYTB2F (5′‐CATTTTGAGGGGCTACAGTTATC‐3′)/CYTB2R (5′‐GTTGGCGGTTAGGGTTCAG‐3′), while the second round of primers was derived from CYTB1F (5′‐CGGCCAAACCCTCGTAGAAT‐3′)/CYTB1R (5′‐GGGCGGAAGGTTATTGTA‐3′). PCR products were subjected to Sanger sequencing (PCR condition in Table S2), and sequences were confirmed by BLAST search in NCBI. All 60 fecal samples were confirmed to be the droppings of black‐necked crane.

### 2.3 Invertebrate diet detection

We adopted the arthropod‐specific primers (ANML) LCO1490 and CO1‐CFMRa (Jusino et al., 2019) targeting the mitochondrial COI gene to characterize the invertebrate diet. PCR was conducted in 25 μL reactions containing 5 μL 5× buffer, 5 μL 5× GC buffer, 2 μL dNTP (2.5 mM), 1 μL forward primer (10 μM), 1 μL reverse primer (10 μM), 0.25 μL Q5 DNA Polymerase, 2 μL DNA template, and 8.75 μL ddH_2_O. The PCR procedures were performed as follows: 2 min at 98°C, followed by 30 cycles at 98°C for 15 s, 55°C for 30 s, and 72°C for 30 s, with a final extension at 72°C for 5 min. PCR products were purified following the manufacturer's instructions.

We constructed the DNA library by using a TruSeq Nano DNA LT Library Prep Kit (Illumina, CA, USA) and a purified library using VAHTS DNA Clean Beads (Vazyme, Nanjing, China). Library quality was quantified using a QuantiFluor fluorescence quantitative system (Promega, Beijing, China), and a library whose concentration fulfilled 20 ng/μL was used for paired sequencing. Paired sequencing was performed on an Illumina NovaSeq platform at Personalbio (Shanghai Personal Biotechnology, Nanjing, China). Samples that failed in PCR amplification were removed (five in pre‐breeding and six in post‐breeding), resulting in 49 samples for data processing (Table S1).

Data processing was performed as previously described (Zhu et al., 2024), except that we used a 97% clustering approach in this study. Sequencing data (4,796,756 raw reads) were processed using the EasyAmplicon pipeline (Liu, Chen, et al., 2023). We used “fastx_filter” in VSEARCH (v2.14.1) for primer cutting and quality control, with an error rate of 0.01, and “cluster_otus” in USEARCH (v10.0.240) for clustering. Dereplication was conducted using “derep_fullength,” with a minimum unique size of 20. “usearch_global” in VSEARCH was used to generate an operational taxonomic unit (OTU) table.

Sequences were searched against the National Center for Biotechnology Information database (NCBI_2019.10), and the top hit was retained, resulting in 1226 OTUs. Hits with a minimum of 90% similarity (*n* = 154) were retained. Non‐target sequences were searched against the Barcode of Life Data Systems database for further taxonomic confirmation (Nell et al., 2023). Any non‐dietary data, such as for the black crane itself, vertebrates, plants, and parasites, were removed. After filtering, 1,852,075 unannotated and low similarity reads were removed, resulting in 94 OTUs with 2,944,681 reads.

### 2.4 Statistical analysis

#### 2.4.1 Alpha diversity, beta diversity, and relative abundance of diet

All statistical analyses were performed using R version 4.2.3 (R Foundation for Statistical Computing, Vienna, Austria). Three alpha diversity indexes (Richness, Shannon, and Simpson) and two beta diversity indexes (Bray–Curtis and Jaccard distances) were calculated using the “vegan” package at the OTU level and genus level. We estimated seasonal effects based on the two distances using the analysis of similarities (ANOSIM) function with 999 permutations in the “vegan” package. Non‐metric multidimensional scaling (NMDS) analyses were conducted using the “metaMDS” function in the “vegan” package. We adopted linear mixed models with sampling season as a fixed factor and sampling location as a random factor using “lme4” v1.1.33 (Bates et al., 2014). We conducted transformations using the “powerTransform” function in “car” v3.1.2 (Fox et al., 2007), without conforming to the normality or constant variance of the model residuals. To assess the relative abundance of the taxa of interest, we used generalized linear mixed models with Dirichlet distribution in the “brms” package, accounting for the sum constraint of the proportions. Sampling season was considered a fixed factor, whereas sampling location was a random factor.

#### 2.4.2 Analysis of differential invertebrate diet between seasons

We estimated the differences in OTU levels between the pre‐ and post‐breeding seasons using a negative binomial generalized linear model in the “edgeR” package (Robinson et al., 2010). To find robust differential taxa between seasons, OTUs with relative abundance >0.01% were retained. *p*‐Values were corrected for multiple tests using the Benjamin and Hochberg method (Narkevich et al., 2020). OTUs were significantly enriched or depleted if they had an adjusted *p*‐value < .05 and |log_2_FC| > 2.0.

We adopted random forest models with machine learning algorithms to identify distinct features between seasons using the R package “randomForest” (Breiman, 2001). We utilized 45 samples (about 90% of the dataset) as the training set to construct the seasonal classification model. First, we estimated error rates for phylum, order, family, genus, and OTU level to select the taxon level with the lowest error rate with default parameters. We selected important features using the “rfcv” function based on the cross‐validation error curve from five trials.

We performed linear discriminant analysis (LDA) effect size (LEfSe) (Chang et al., 2022) to identify seasonal changes in dietary composition at the phylum, order, family, and genus levels simultaneously using an online tool (https://www.bic.ac.cn/BIC/#/). Non‐parametric Kruskal–Wallis rank sum tests were used to identify species with significant differences in abundance between subgroups. Wilcoxon rank sum tests were then used to assess the consistency of differences among species across intergroup subgroups. Linear regression analyses were used to estimate the magnitude of changes in the abundance of each component (species) and determine statistically significant diets. We considered the taxonomic categories with log_10_ LDA scores >3 and *p* < .05 to find more robust different features between groups, as described in *Nature Communications* (Yao et al., 2017), *Molecular Ecology* (Lu et al., 2023), and the *ISME Journal* (Amato et al., 2019).

#### 2.4.3 Dietary niche of black‐necked cranes between seasons

We calculate the niche width of black‐necked cranes in different seasons using the R package “spaa” v0.2.2 (Zhang & Zhang, 2013).

## 3 RESULTS

### 3.1 Pre‐ and post‐breeding dietary differences in frequency of occurrence

We obtained 4,796,756 high‐quality reads based on ANML metabarcoding. Rarefaction analysis revealed that the sequencing data captured most diet components from each black‐necked crane fecal sample (Figure S2). We identified 3 phyla, 7 classes, 13 orders (pre‐breeding: 12, post‐breeding: 2), 21 families (pre‐breeding: 17, post‐breeding: 4), 38 genera (pre‐breeding: 22, post‐breeding: 18), and 47 species (pre‐breeding: 27, post‐breeding: 25) in the invertebrate diet of breeding black‐necked cranes, with 93.6% annotation at the genus level.

At the order level, Diptera was observed in all samples, and 11 unique orders and one unique order (Orthoptera) were investigated in the pre‐ and post‐breeding periods, respectively (Table S3). At the family level, Tipuilidae and one classified Diptera family were observed in both seasons. Fifteen and four unique families were detected in the pre‐ and post‐breeding periods, respectively (Table S4).

Within 38 genera, we found 20 and 16 unique genera in the pre‐ and post‐breeding periods, respectively, and two shared genera *Tipula* and *no rank_Diptera* sp. *BOLD:ACY6082* in both seasons (Table S5). *Tipula* prey was detected in all samples, and the other 10 genera of prey occurred in >10% of samples (Figure S3, Table S5). Furthermore, we observed nine and seven arthropod genera with a prevalence of 10% in the pre‐ and post‐breeding periods, respectively. The other most prevalent genera, Stethophyma (frequency 54.2%), besides *Tipula*, was observed in post‐breeding.

### 3.2 Relative abundance of dominant diets between pre‐ and post‐breeding seasons

We identified arthropod, mollusk, and annelid phyla, with arthropods comprising 98.74% of the total relative abundance across the two seasons (Table S6).

Diptera has the highest mean relative abundance at the order level (82.17% and 95.61% of pre‐ and post‐breeding samples, respectively). The relative abundance of the other four orders, Lepidoptera, Hymenoptera, Orthoptera, and Coleoptera, was >1% (Table S7).

**FIGURE 2:**
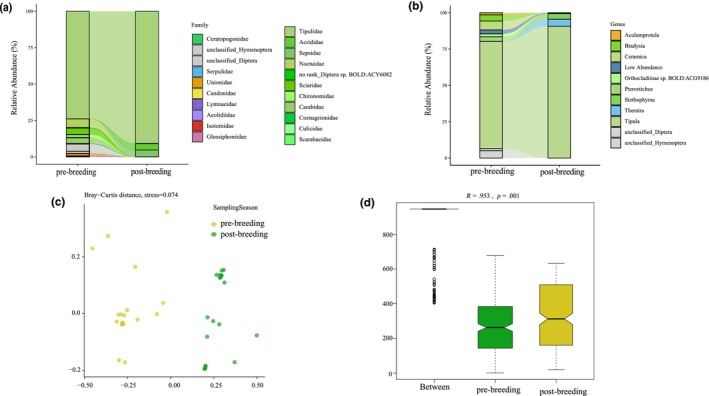
Seasonal invertebrate diet variation in black‐necked crane. (a) Mean relative abundance of prey items in pre‐ and post‐breeding seasons at family level. (b) Mean relative abundance of prey items (top 10) in pre‐ and post‐breeding seasons at genus level. (c) Bray–Curtis distance for seasonal invertebrate diet with NMDS. (d) Between‐ and within‐group differences based on Bray–Curtis distance.

Among the 21 families, eight families exhibited a mean relative abundance >1%, with Tipulidae having the highest mean relative abundance (82.09%, Figure 2a, Table S8). At the genus level, eight of the 38 prey items showed a mean relative abundance of >1%, with *Tipula* predominating (82.09%) across seasons (Figure 2b, Table S9).

However, we did not observe a significant difference in invertebrate relative abundance between the pre‐ and post‐breeding periods for 3 phyla, 13 orders, and the top 10 families and genera (Tables S6–S9).

### 3.3 Invertebrate diet diversity in breeding black‐necked crane

At MOTU level, in terms of alpha (within‐sample) diversity, the pre‐ and post‐breeding seasons differed in terms of the richness index (observed OTUs, *χ* ^2^ = 4.3903, *p* = .036, Figure S4) but not the Shannon and Simpson indexes (*χ* ^2^ = 290, *p* > .05 and *χ* ^2^ = 0, *p* > .05, respectively; Figures S5a and S6a). Black‐necked cranes preyed on more invertebrate items during the post‐breeding than pre‐breeding period. Between‐sample diversity (beta diversity) showed no difference between seasons based on Bray–Curtis distances (*χ* ^2^ = 0.1145, *p* = .735, Figure S7a) but differed between seasons based on Jaccard distances (*χ* ^2^ = 12.172, *p* < .001, Figure S8a). Pre‐breeding samples had a higher beta diversity compared to post‐breeding samples based on Jaccard distances. Invertebrate diet composition varied and was mainly clustered by season through axis one (stress = 0.074, *R* = .953, *p* = .001, Figure 2c,d). The pre‐breeding samples were more dispersed than post‐breeding samples.

However, at the genus level, black‐necked cranes tended to have more diversity during pre‐breeding than post‐breeding (Figures S5b and S6b; Shannon: *χ* ^2^ = 0.4729, *p* = .492, Simpson: *χ* ^2^ = 0.8649, *p* = .352). Beta diversity in the pre‐breeding period tended to be higher than that in the post‐breeding samples (Figures S7b and S8b; Bray–Curtis: *χ* ^2^ = 0.290, *p* = .590, Jaccard: *χ* ^2^ = 0.513, *p* = .479). Invertebrate diet composition at the genus level was also mainly clustered by season (stress = 0.074, *R* = .036, *p* = .024).

### 3.4 Differential invertebrate diet between pre‐ and post‐breeding seasons

**FIGURE 3:**
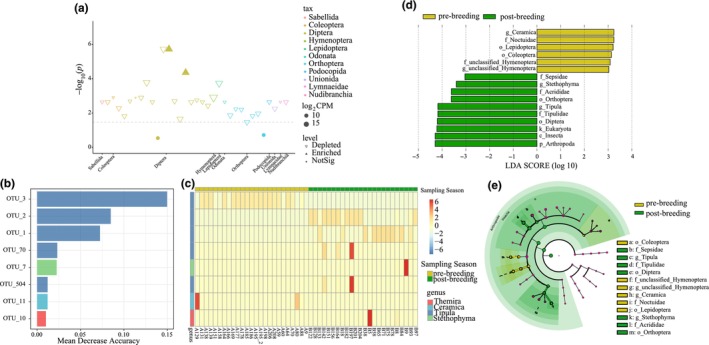
Invertebrate diet comparison between pre‐ and post‐breeding. (a) Enriched and depleted OTUs between pre‐ and post‐breeding period. The control represents pre‐breeding samples. (b) Top eight OTUs (based on relative abundance) detected in the training set using random forest models. (c) Relative abundance of eight OTUs from random forest models for all samples. (d) Discriminant prey features from LDA test. Taxa with log_10_ (LDA) >3 are shown. (e) Cladogram of prey items from LDA test with concentric circles representing the taxonomic level from phylum to genus, moving from the inner OTU.

To reveal the breeding season‐related changes in invertebrate diet, we compared the raw abundances of 34 OTUs (relative abundance >0.01%) between the pre‐ and post‐breeding periods. We detected 30 depleted and two enriched OTUs in the post‐breeding season, with most of the different OTUs coming from Diptera and Orthoptera (Figure 3a). Only two Diptera OTUs were not significant between seasons.

We analyzed the seasonal differences in diet‐related features. Our random forest models revealed that OTUs showed the highest accuracy of dietary classification within phylum, class, order, family, genus, and OTU levels (Table S10). The cross‐validation error rate was 0.031 when adopting the eight most relevant OTUs—defined as differential diet taxa in this study (Table S11, Figure 3b). The model accurately predicted the outcomes of the five samples from the test set data (Table S12). Eight OTUs differed between seasons (Table S13). The pre‐breeding samples were enriched in OTU_3 and OTU_11, while the post‐breeding samples had the highest relative abundance of the other six OTUs (Figure 3c, Figure S9).

Linear regression analysis revealed 16 discriminant features at different taxonomic levels, with 6 and 10 enriched during the pre‐ and post‐breeding periods, respectively (Figure 3d). The more abundant features in the pre‐breeding period were associated with Lepidoptera and Coleoptera, while those in the post‐breeding period were associated with Diptera and Orthoptera (order level); and Tipulidae, Sepsidae, and Acrididae (family level) (Figure 3e).

### 3.5 Dietary niche of black‐necked cranes between seasons

The wider niche width of the pre‐breeding black‐necked cranes also revealed a more varied invertebrate diet before breeding at species and genus levels (Figure S10).

## 4 DISCUSSION

Understanding an animal's diet is important for understanding their foraging ecology. We investigated the invertebrate diet of black‐necked cranes during breeding in the highland Zoige wetland and explored their dietary shifts from the pre‐breeding to post‐breeding periods.

### 4.1 Preferences in the black‐necked crane invertebrate diet across breeding seasons

We identified 13 orders, 21 families, and 38 genera among the invertebrates consumed by black‐necked cranes during the breeding season, which covered more taxa and provided greater resolution in comparison to a previous study based on microscopic examination (five orders) (Liu et al., 2019). We adopted a similar timescale to the previous study to promote the comparability of the two studies and detection methods. Orthoptera, Unionida, or Entomobryomorpha may not have been previously detected by microscopic examination because they were digested (Liu et al., 2019). In the present study, the dominant order was Diptera, regardless of relative abundance or frequency (100%), while Coleoptera was the predominant food item in the other study (Liu et al., 2019). This discrepancy is likely due to the difficult detection of Diptera adults and larvae using visual techniques.

We observed prevalence for Diptera (mainly *Tipula*) during the pre‐ and post‐breeding seasons, suggesting dietary composition is likely related to the accessibility, nutritional value, and manipulability of these animals (Aguirre et al., 2003; Ayala‐Berdon et al., 2023; Józefiak et al., 2016; Prado e Castro & Ameixa, 2021). Soil arthropods, such as Diptera, thrive in areas with high precipitation, mild climates, and high plant diversity (Fischer et al., 2022; Zhao et al., 2017), which coincides with the habitat preferences of the black‐necked cranes. This finding is consistent with observations on Indiana bats and Sichuan partridges during the rainy season (O'Rourke et al., 2021; Tang et al., 2023).

The high occurrence of Dipterans in the black‐necked crane diets indicates a preference for more easily digestible energy‐rich soft‐bodied prey. Diptera are rich in high‐quality proteins and bioactive compounds, such as polyunsaturated fatty acids and antimicrobial peptides, and offer enough energy to cater for the high‐calorie demands of pre‐breeding preparations and winter migrations (Józefiak et al., 2016; Prado e Castro & Ameixa, 2021). Prey hardness is known to significantly influence predatory behavior (Aguirre et al., 2003; Ayala‐Berdon et al., 2023).

### 4.2 Seasonal variation in invertebrate prey by black‐necked cranes

Seasonal changes in resource availability led to dietary variation in animals (De Camargo et al., 2014; Norconk, 1996; Panaino et al., 2022). Similarly, our findings revealed that black‐necked cranes consumed distinct invertebrate taxa between the two seasons, suggesting a seasonal dietary pattern. The black‐necked crane's dietary choices fit optimal foraging theory, which states that during the season of abundant food, the increase in favorable food causes the animal to switch from a diversified diet to a single, favorable diet (MacArthur & Pianka, 1966). Higher precipitation and temperatures tend to promote productivity during the May–September growing season (Guo et al., 2018). Furthermore, soil arthropod abundance is positively related to plant species richness (Zhao et al., 2017).

The difference between the pre‐ and post‐breeding diets in black‐necked cranes might be due to both seasonality in arthropod occurrence and seasonality in arthropod abundance. For example, Acrididae were observed only in post‐breeding samples, which might be due to their phenology. Additionally, the hatching of Acrididae eggs has temperature dependence and might not be possible until at least 14°C (Fielding, 2011), and the inability to provide suitable temperature conditions during the pre‐breeding might be why Acrididae could not be detected in the pre‐breeding. However, Scarabaeidae and Carabidae, two families detected in only pre‐breeding samples, were present in non‐growing and growing seasons in the Qinghai‐Tibetan Plateau as described in the literature (Lu et al., 2024; Tan et al., 2013; Wang et al., 2024), suggesting that those species differed in abundance, not in occurrence. According to these previous studies and our findings, black‐necked cranes might experience prey choice when they face same invertebrate at the two seasons (Lu et al., 2024; Tan et al., 2013; Wang et al., 2024). In pre‐breeding, when food resource quantities are scarce, black‐necked cranes will widen the area of food collection to ensure maximum energy intake (Dong et al., 2024; Li et al., 2023). The diet niche of black‐necked cranes in the early breeding period was wider (Figure S9). This flexible pattern has been reported in other species, such as rhesus macaque, dhole (Steinmetz et al., 2021), chacma baboon (Johnson et al., 2013), and gorilla (Rothman et al., 2007), revealing that animals have highly rich diets during seasons with food shortages.

Acrididae, one of the main foods of the black‐necked crane in post‐breeding, plays an important role in nutrient cycling and is an important part of the ecosystem (Song et al., 2018). Most Acrididae species are believed to be related to grasslands, but some species live around aquatic plants (Le Gall et al., 2019; Song et al., 2018).

More specifically, three *Tipula* OTUs, OTU_1, OTU_2, and OTU_3, showed distinct relative abundances between seasons and were able to discriminate the seasonal change in diet (Figure 3a,b). *Tipula* (common crane flies) contains over 2000 species (Hofsvang et al., 2019) worldwide that are active throughout the year. The nine *Tipula* OTUs identified in this study belong to two known species (*Tipula paludosa* and *Tipula dorsimacula*) and three unknown species (*Tipula* sp. *BOLD. AAF8981, Tipula* sp. *SOKN048, and Tipula* sp. *BIOUG02372‐H07*). Since *Tipula* species have similar morphological characteristics, such as long bodies and legs, and may have similar nutritional qualities, the seasonal preference for certain *Tipula* species may be related to their occurrence. The low occurrence of Orthoptera in the black‐necked crane diet was also likely due to their limited occurrence during the non‐growing season. Further studies on Arthropoda ecology in Tibetan Plateau wetlands are needed to elucidate the dietary selection dynamics in the black‐necked crane.

### 4.3 Importance of invertebrate diet for black‐necked cranes

Breeding season is an important period in the black‐necked crane population, and abundant energy intake has important effects on the reproductive population of black‐necked cranes (Cheng & Ma, 2023). Arthropods are an important part of the wetland ecological environment, which is sensitive to environmental changes (Batzer & Wu, 2020). Studies have revealed that wetland degradation reduced arthropod abundance (Majeed et al., 2020), which might result in a shortage of food resources and negatively affect wetland‐dependent birds (Nell et al., 2023), such as the black‐necked crane. Thus, the arthropod diet of black‐necked cranes requires further research for the conservation of black‐necked crane populations (Nell et al., 2023).

Measures to protect wetlands (e.g., expanding the scope of protected areas to reduce disturbances caused by human activities and restoring degraded wetlands) might not only increase the number of waterfowl habitats but also improve their habitat quality (Beatty et al., 2014). Because birds in high‐quality breeding grounds (with rich food resources) have a higher reproduction rate than those in low‐quality breeding grounds (with poor food resources) (Verhulst & Nilsson, 2008), protecting wetlands will indirectly increase the crane population.

For animals in a changing environment, understanding the interrelationship between diet and food availability is necessary to improve the guidance for conservation efforts. Based on dietary habits, the main food distribution area in threatened species should be considered a priority for protection. For example, Carabidae are important predators in wetlands and the dominant diet of black‐necked cranes, which consume aestivating aquatic invertebrates (Batzer & Wu, 2020). Degradation of the wetland will affect the survival of Carabidae and further affect black‐necked cranes (Batzer & Wu, 2020). Seasonal diet research has been conducted for several threatened species and could be useful for their future protection; examples of these species are the astern quoll (*Dasyurus viverrinus*) (Fancourt et al., 2018), Sichuan snub‐nosed monkey (*Rhinopithecus roxellana*) (Li, 2006), Italian hare (*Lepus corsicanus*) (Freschi et al., 2016), and Red‐headed Wood Pigeon (*Columba janthina nitens*) (Ando et al., 2016). Our findings on the seasonal dietary composition of black‐necked cranes will facilitate the timely assessment of the survival status of black‐necked cranes during seasonal transitions.

## 5 CONCLUSION

This study investigated invertebrate diet composition during pre‐ and post‐breeding seasons in the large wetland black‐necked crane. We observed seasonal patterns in invertebrate occurrence by assessing diet. Our findings elaborate on the diet variation in black‐necked cranes to seasonal changes in resources. Tipula predominated across seasons. We observed 20 and 16 unique genera in the pre‐ and post‐breeding periods and an obvious seasonal pattern in food composition and dietary structure. In response to seasonal fluctuations from pre‐breeding to post‐breeding seasons, black‐necked cranes tended to have less within‐sample diversity and lower between‐sample heterogeneity at the genus level. Black‐necked cranes had a wider dietary niche in the pre‐breeding period. The most important diet for discriminating seasonal changes in the invertebrate diet of black‐necked crane was Tipula. The seasonal pattern observed in this study might be due to seasonality in arthropod phenology and seasonality in arthropod abundance, which might suggest a potential prey choice in black‐necked cranes. Our comprehensive investigation of invertebrate diet dynamics also elaborates on the foraging ecology of the black‐necked crane in its rapidly changing highland home. Future research should focus on the broader ecological impact of black‐necked crane foraging behavior on highland ecosystems, including the potential effects on local arthropod populations, vegetation dynamics, and overall ecosystem functioning.

## AUTHOR CONTRIBUTIONS

**Ruifeng Ma:** Formal analysis (equal); validation (equal); visualization (equal); writing – original draft (equal); writing – review and editing (equal). **Shujuan Ma:** Data curation (equal); formal analysis (equal); writing – original draft (equal). **Hongyi Liu:** Conceptualization (supporting); writing – review and editing (equal). **Lei Hu:** Conceptualization (supporting); writing – review and editing (equal). **Yudong Li:** Conceptualization (supporting); investigation (equal). **Ke He:** Visualization (equal); writing – review and editing (equal). **Ying Zhu:** Conceptualization (lead); funding acquisition (lead); project administration (lead); supervision (lead); visualization (equal); writing – original draft (equal); writing – review and editing (equal).

## CONFLICT OF INTEREST STATEMENT

All the authors declare that they have no conflict of interest.

## Supporting information


Figures S1–S10

Tables S1–S13

## Data Availability

The raw sequence data reported in this paper have been deposited in the Genome Sequence Archive (Genomics, Proteomics & Bioinformatics 2017) in the National Genomics Data Center (Nucleic Acids Res 2021), China National Center for Bioinformation/Beijing Institute of Genomics, and Chinese Academy of Sciences, under accession number CRA013742 (https://ngdc.cncb.ac.cn/gsa/s/VR7Z7JRa).
