# Supplementary material for: Seasonal changes in invertebrate diet of breeding black‐necked cranes (Grus nigricollis)
Source: Ecol Evol. 2024 Aug 29;14(9):e70234. doi: 10.1002/ece3.70234 (PMC11362503; doi:10.1002/ece3.70234)
Supplement: Supplementary file 1 — Figures S1–S10 [file ECE3-14-e70234-s001.docx]

**Figure S1** Represent fecal samples collected during the study period. (a) black feces in spring; (b) black feces in autumn; (c) white and black feces in spring; (d) black and white feces in autumn; (e) brown samples in spring; (f) brown samples in autumn; (g) white feces in spring; (h) white feces in autumn; (i) gray feces in spring; (j) gray feces in autumn; (k) red feces in spring; (l) sorrel samples in autumn; (m) green feces in spring; (n) green feces in autumn.


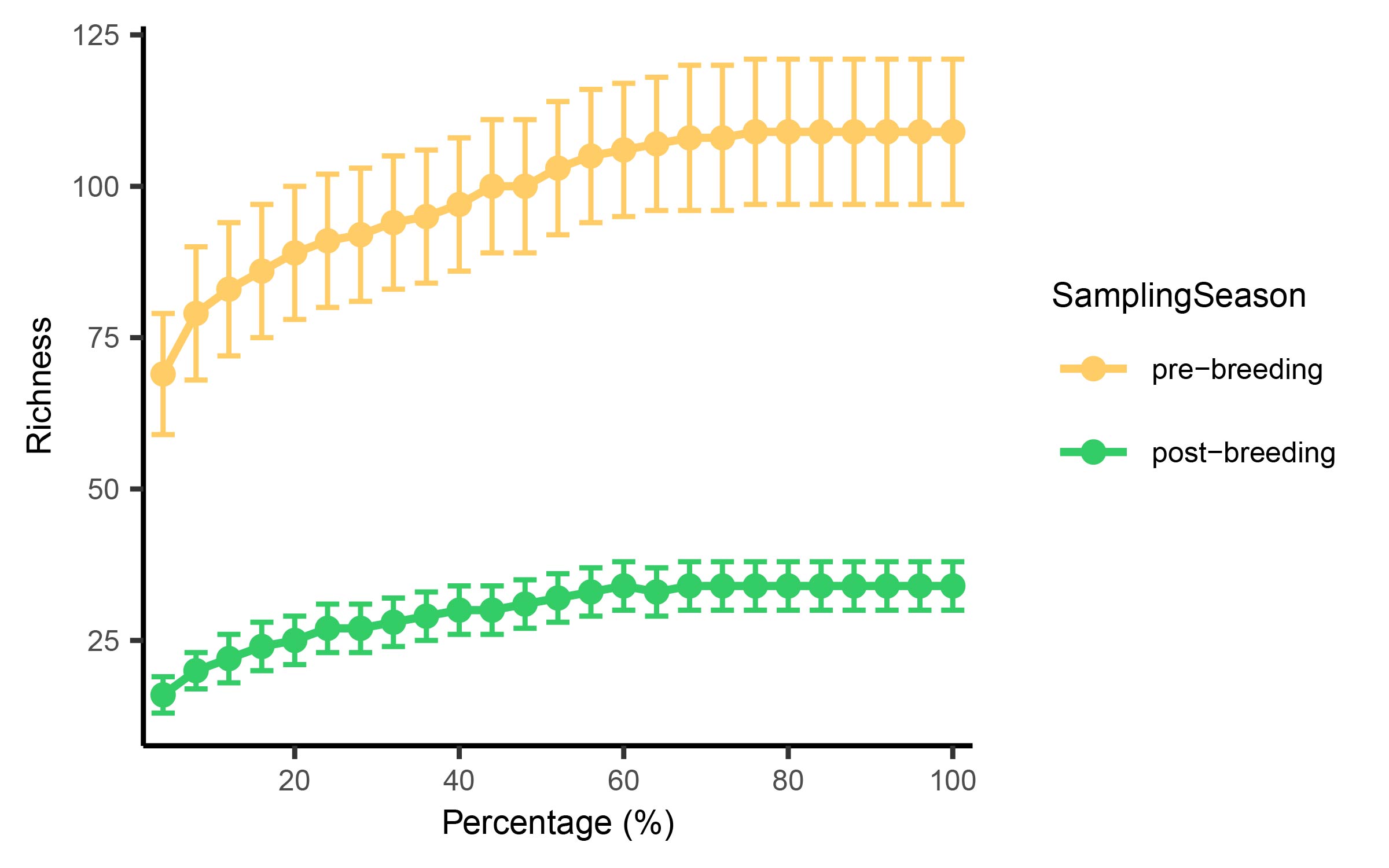


**Figure S2** Coverage of members in the diet by the black neck crane.


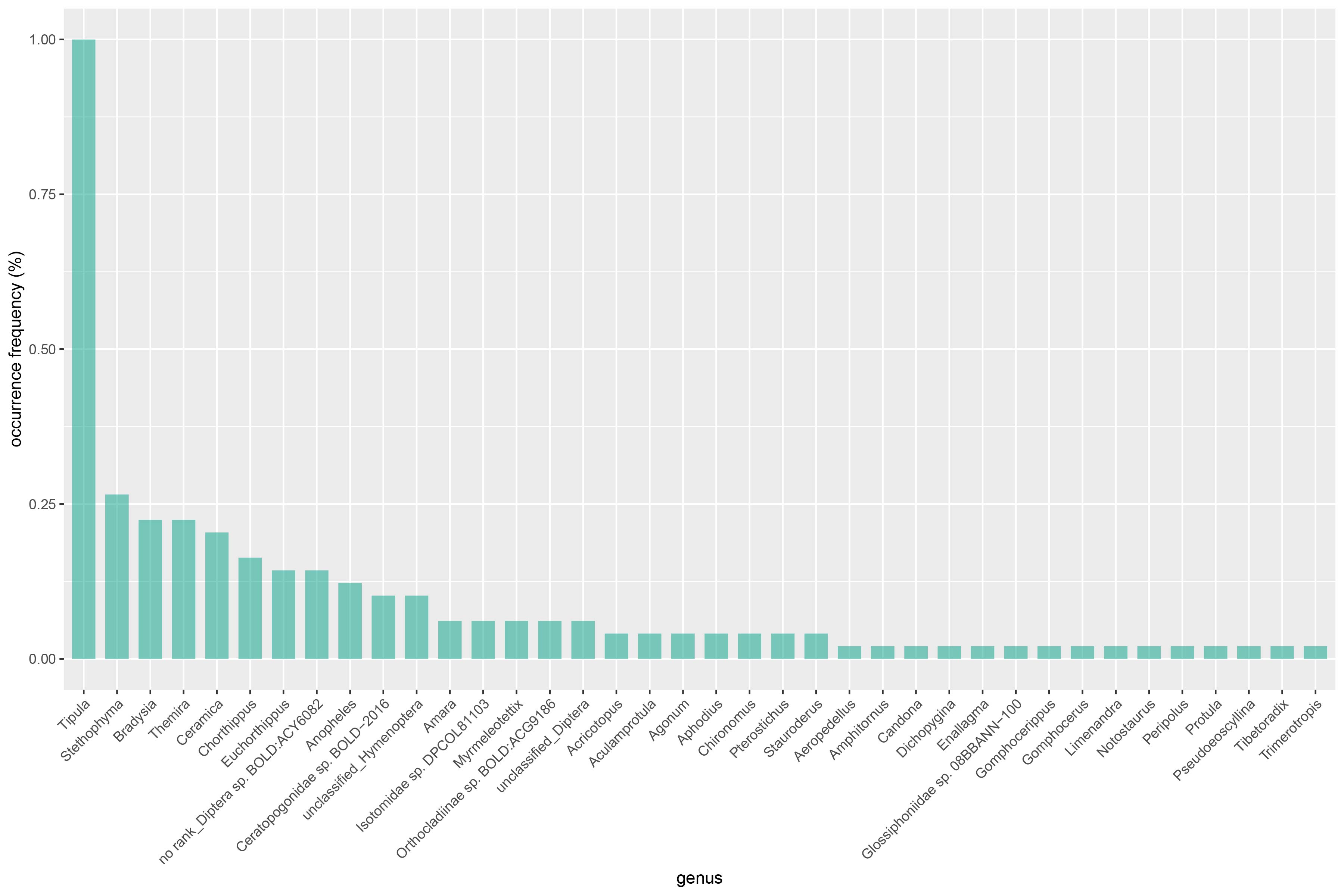


**Figure S3** The occurrence frequency on genus level.


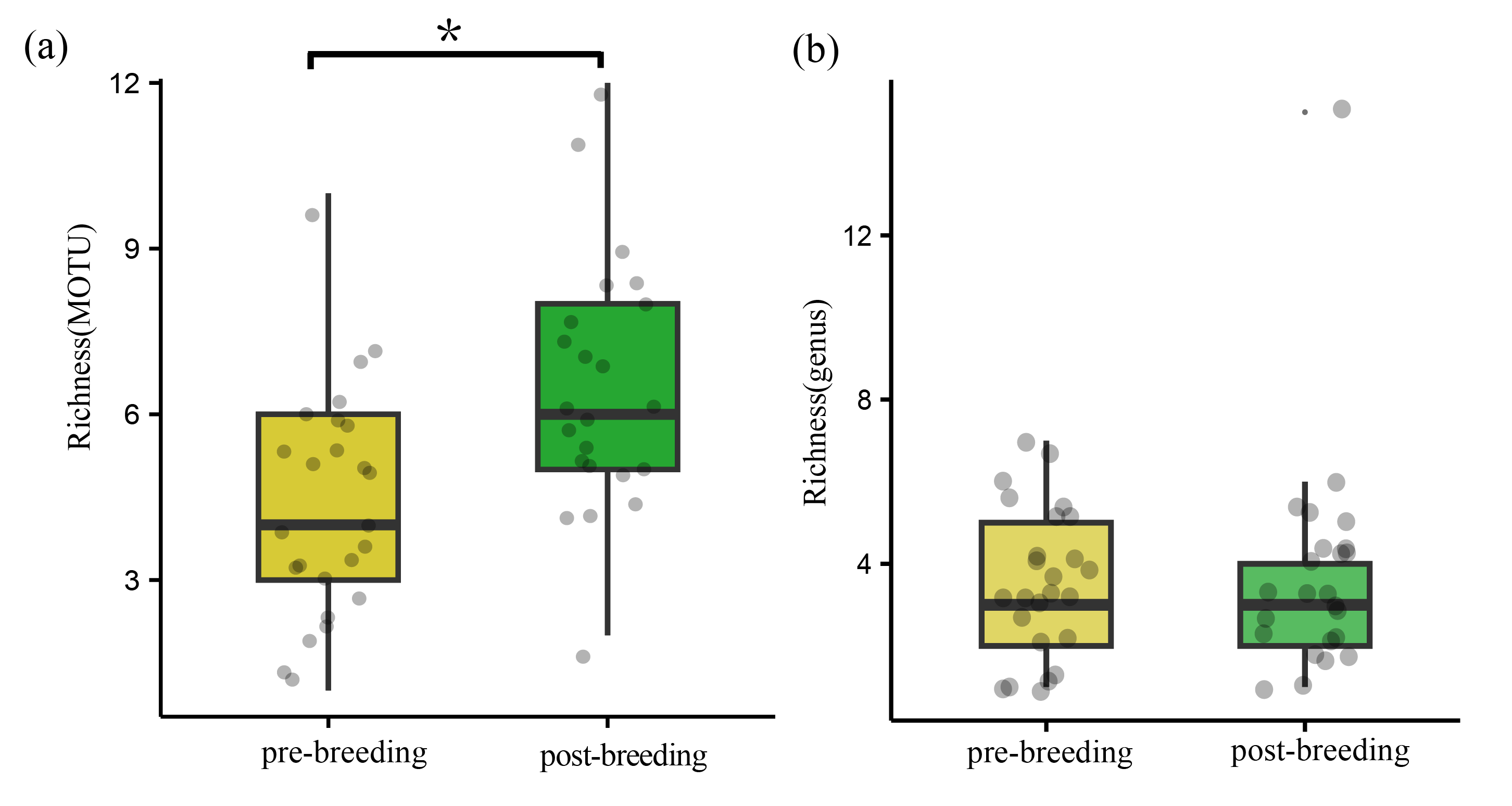


**Figure S4** Richness in two seasons at MOTU and genus level. * *P* < 0.05, ** *P* < 0.001.


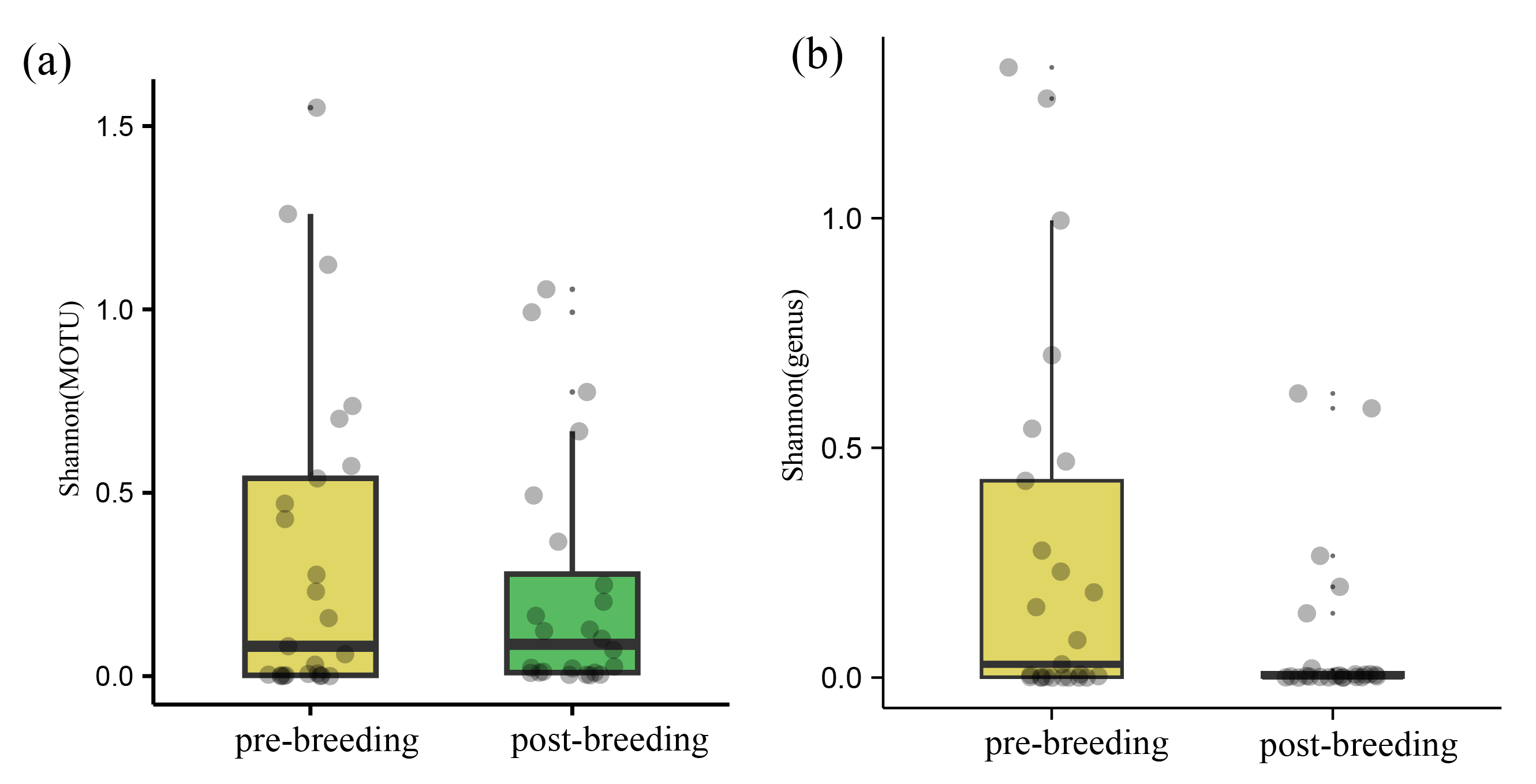


**Figure S5** Shannon in two seasons at MOTU and genus level. * *P* < 0.05, ** *P* < 0.001.


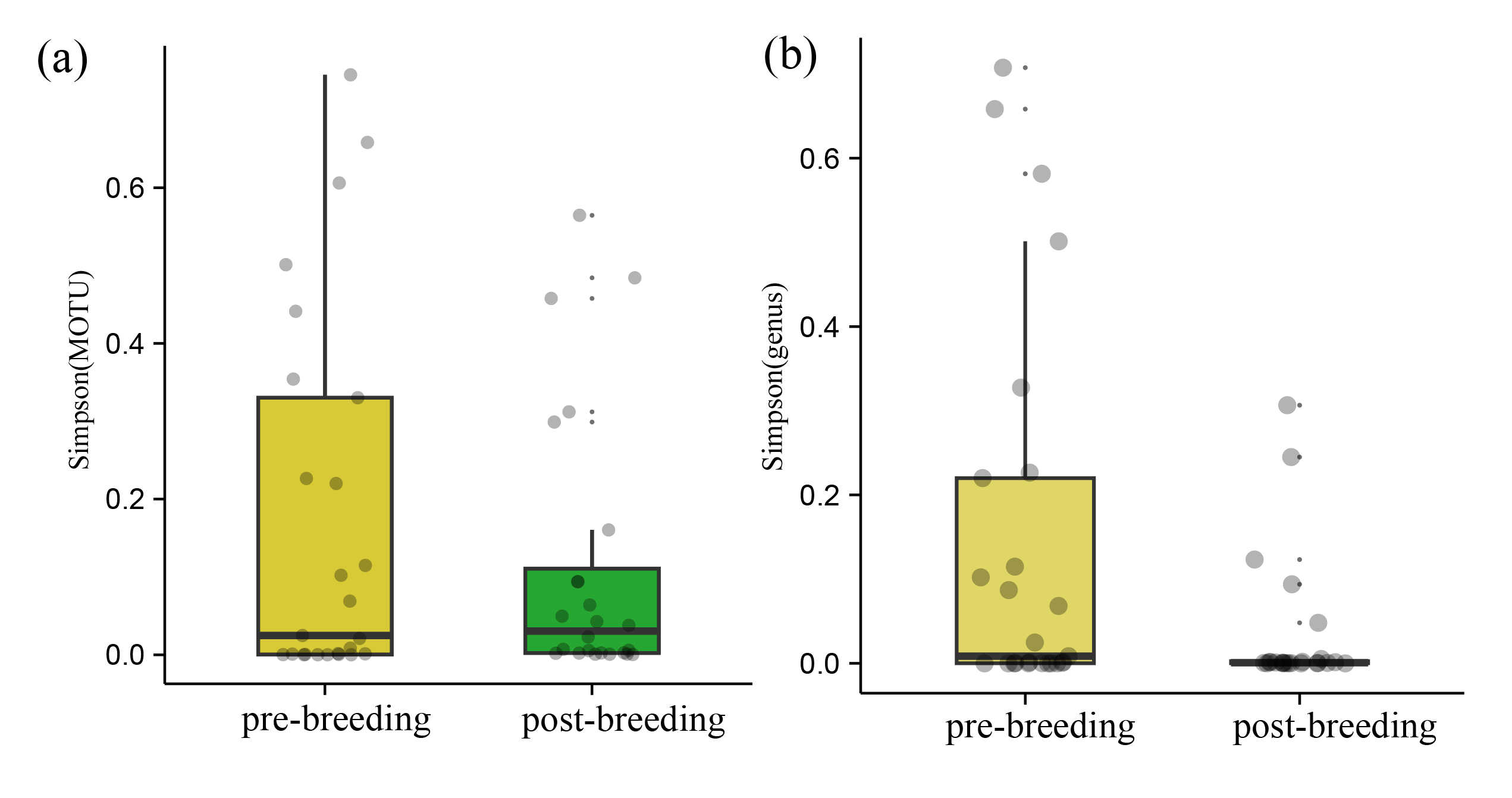


**Figure S6** Simpson in two seasons at MOTU and genus level. * *P* < 0.05, ** *P* < 0.001.


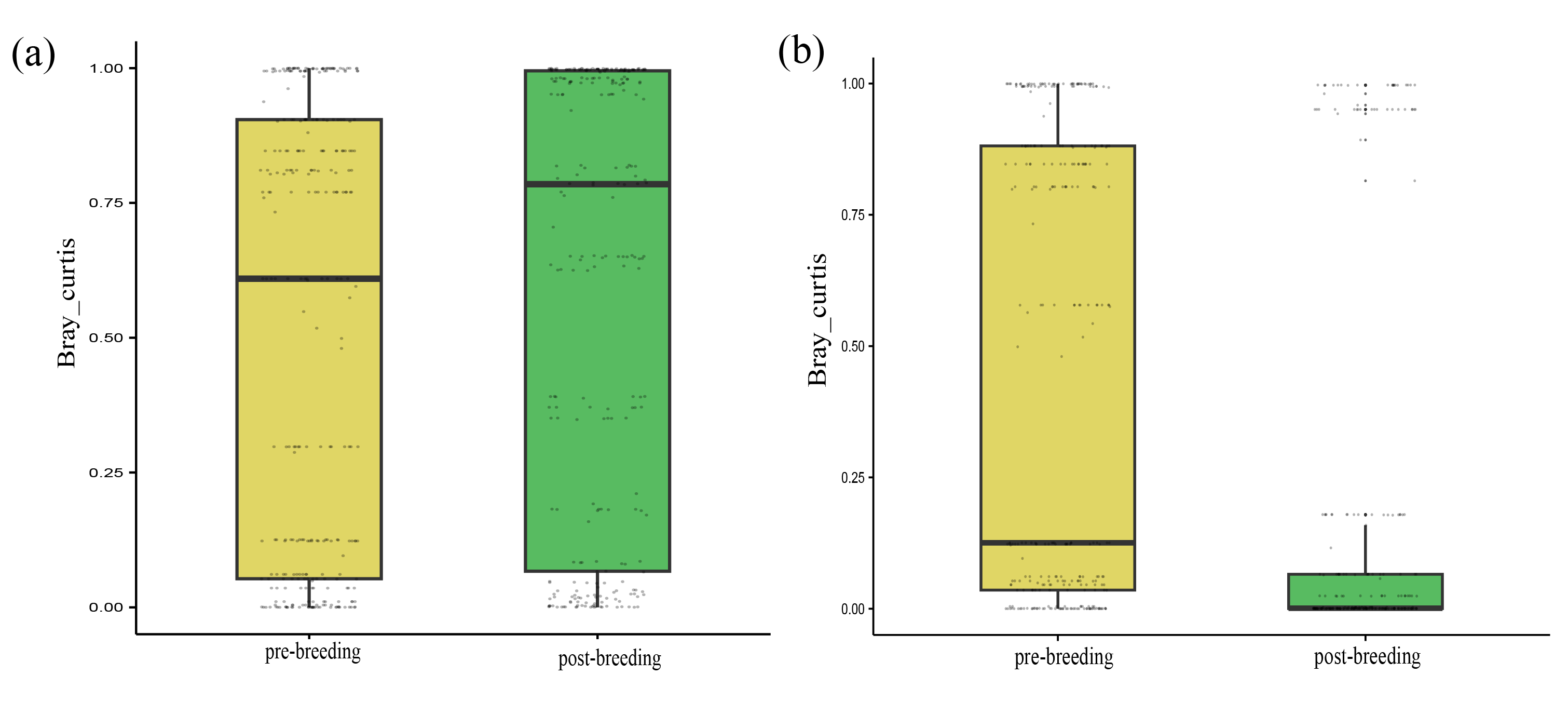


**Figure S7** The Bray_Curtis distance in two seasons at MOTU and genus level. * *P* < 0.05, ** *P* < 0.001.


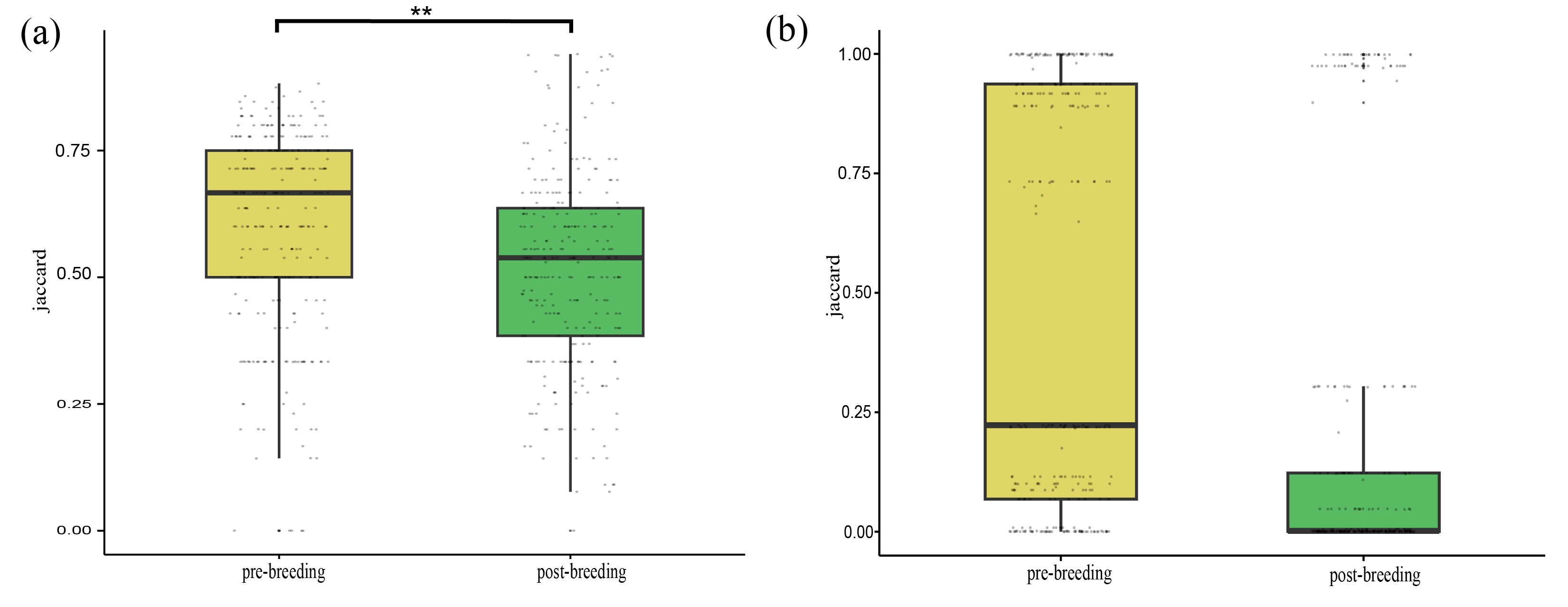


**Figure S8** The Jaccard distance in two seasons at MOTU and genus level. * *P* < 0.05, ** *P* < 0.001.


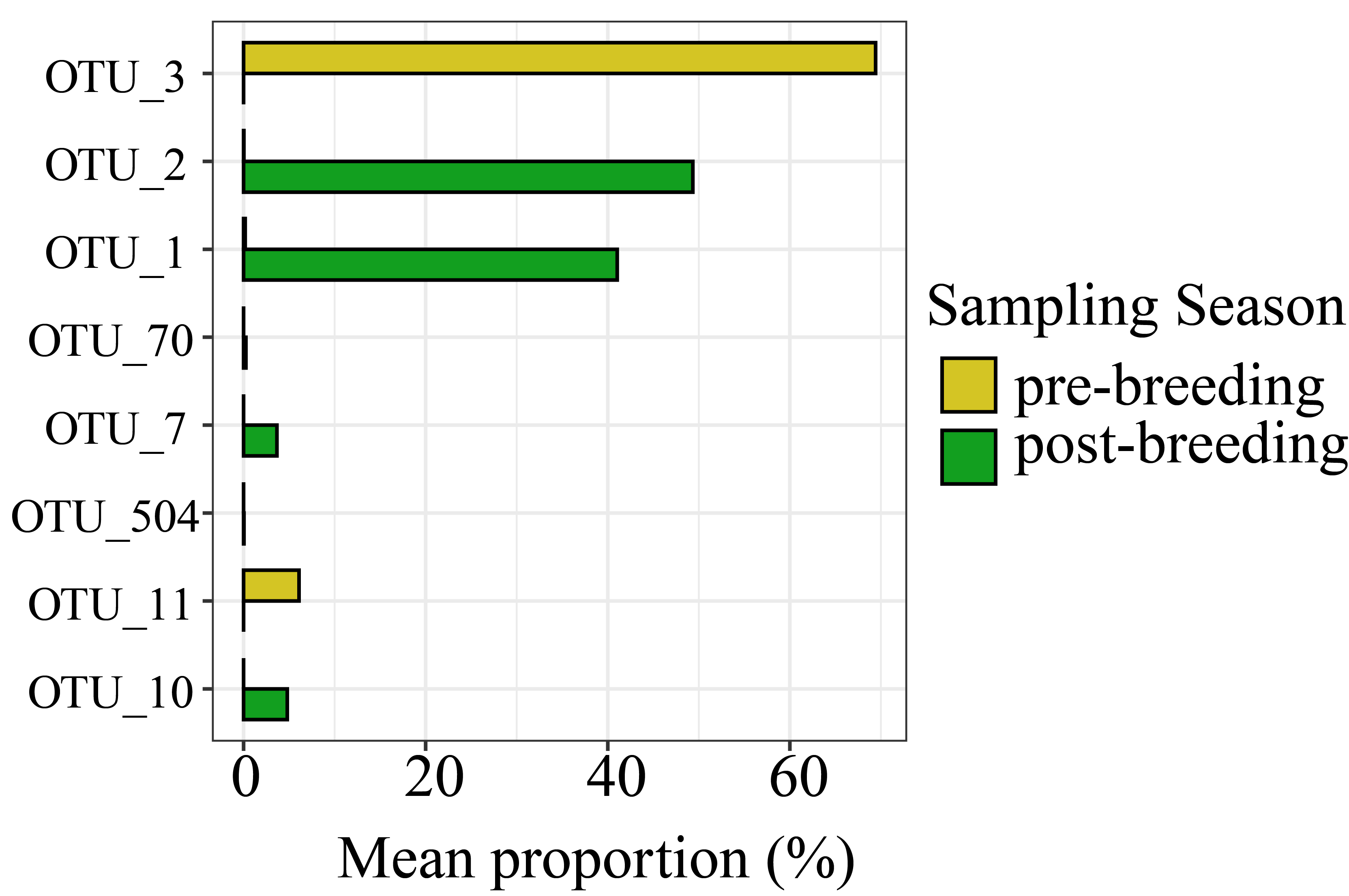


**Figure S9** The relative abundance of eight OTUs between pre- and post-breeding seasons obtained from our random forest model.


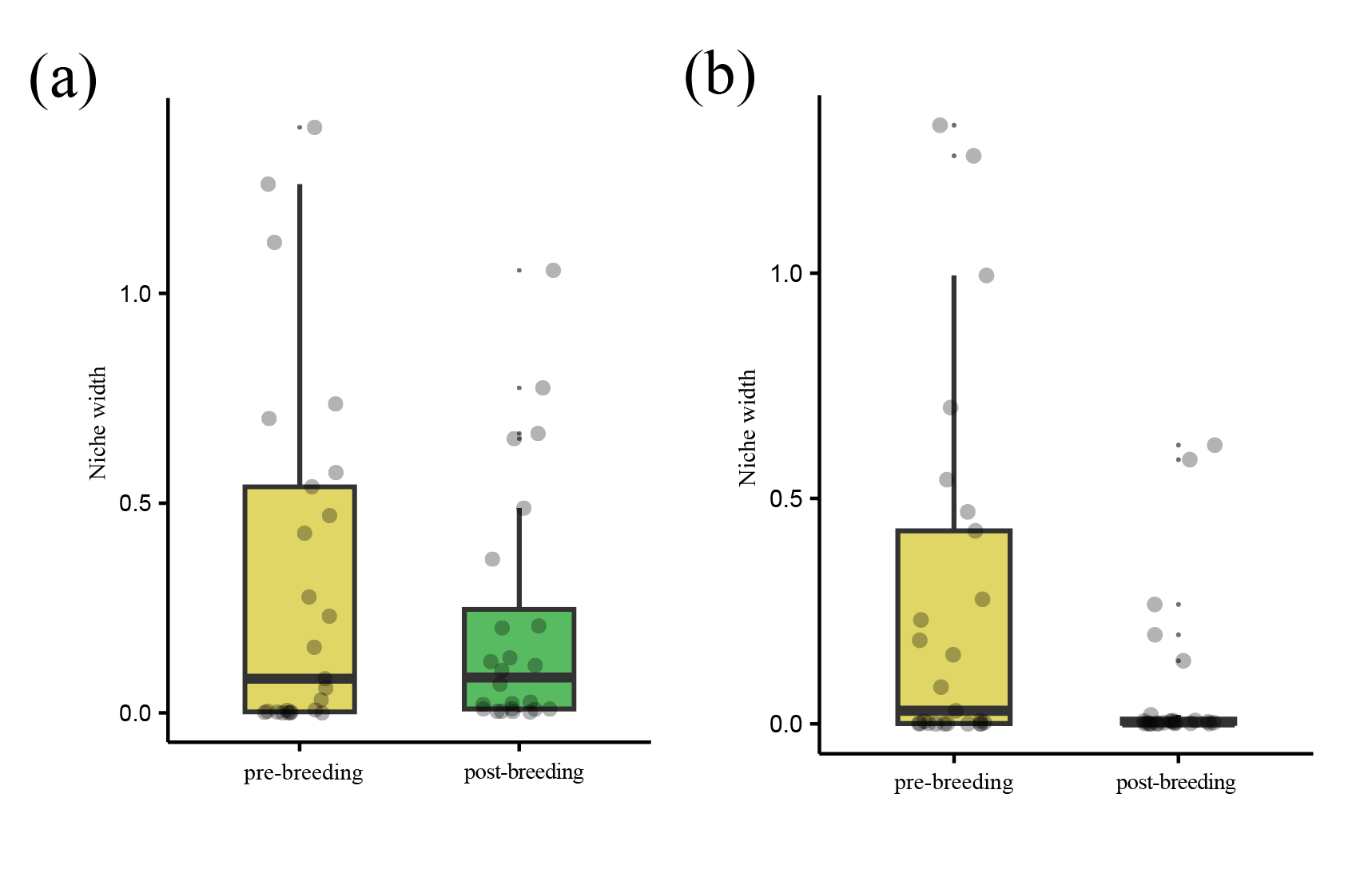


**Figure S10** The niche width of black necked cranes in pre-breeding and post-breeding seasons, (a) species level, (b) genus level.
